# Differential diagnoses of acute ground-glass opacity in chest computed tomography: pictorial essay

**DOI:** 10.31744/einstein_journal/2021RW5772

**Published:** 2021-03-05

**Authors:** Marina Justi Rosa de Matos, Marcela Emer Egypto Rosa, Vanessa Mizubuti Brito, Lucas Tadashi Wada Amaral, Gabriel Laverdi Beraldo, Eduardo Kaiser Ururahy Nunes Fonseca, Rodrigo Caruso Chate, Rodrigo Bastos Duarte Passos, Murilo Marques Almeida Silva, Patrícia Yokoo, Roberto Sasdelli, Gustavo Borges da Silva Teles, Marina Carolina Bueno da Silva, Gilberto Szarf

**Affiliations:** 1 Hospital Israelita Albert Einstein São PauloSP Brazil Hospital Israelita Albert Einstein, São Paulo, SP, Brazil.

**Keywords:** Coronavirus infections, COVID-19, Pandemics, SARS-CoV-2, Thorax/diagnostic imaging, Tomography, X-ray computed, Diagnosis, differential

## Abstract

Ground-glass opacity is a very frequent and unspecified finding in chest computed tomography. Therefore, it admits a wide range of differential diagnoses in the acute context, from viral pneumonias such as influenza virus, coronavirus disease 2019 and cytomegalovirus and even non-infectious lesions, such as vaping, pulmonary infarction, alveolar hemorrhage and pulmonary edema. For this diagnostic differentiation, ground glass must be correlated with other findings in imaging tests, with laboratory tests and with the patients’ clinical condition. In the context of a pandemic, it is extremely important to remember the other pathologies with similar findings to coronavirus disease 2019 in the imaging exams.

## INTRODUCTION

The tomographic pattern of ground-glass opacities is a non-specific finding, and may reflect interstitial thickening, partial filling or partial collapse of the alveoli, increased blood supply or even a combination of these findings. Radiographically, it is defined as an increase in the lung parenchyma density, but with preservation of the bronchovascular markings, differing from consolidation.^(^ ^1^ ^,^ ^2^ ^)^

The causes of ground-glass opacities can be divided into acute and chronic. Among the acute causes are infections (atypical bacterial and viral infections), alveolar hemorrhage, pulmonary edema, diffuse alveolar damage, pulmonary embolism, and some neoplasms.^(^ ^3^ ^-^ ^9^ ^)^

In the context of the pandemic caused by the coronavirus 2 of severe acute respiratory syndrome (SARS-CoV-2), early diagnosis is mandatory to reduce the risk of infection and its spreading.

Despite being a frequent finding in coronavirus 2019 disease (COVID-19), ground-glass opacities are not pathognomonic of coronavirus infection. Therefore, none of the other causes should be neglected in the differential diagnosis. To help determine a suggestive diagnosis from computed tomography (CT) imaging, this finding should be correlated with the other characteristics of the image and the patients’ clinical and laboratory data.^(^ ^10^ ^)^ The objective of this study was to review and help differentiate pathologies with ground-glass opacities in the lung parenchyma on CT, through a literature review based on tomographic images from our service.

## DIFFERENTIAL DIAGNOSIS OF GROUND-GLASS OPACITIES

### Alveolar hemorrhage

**Figure 1:**
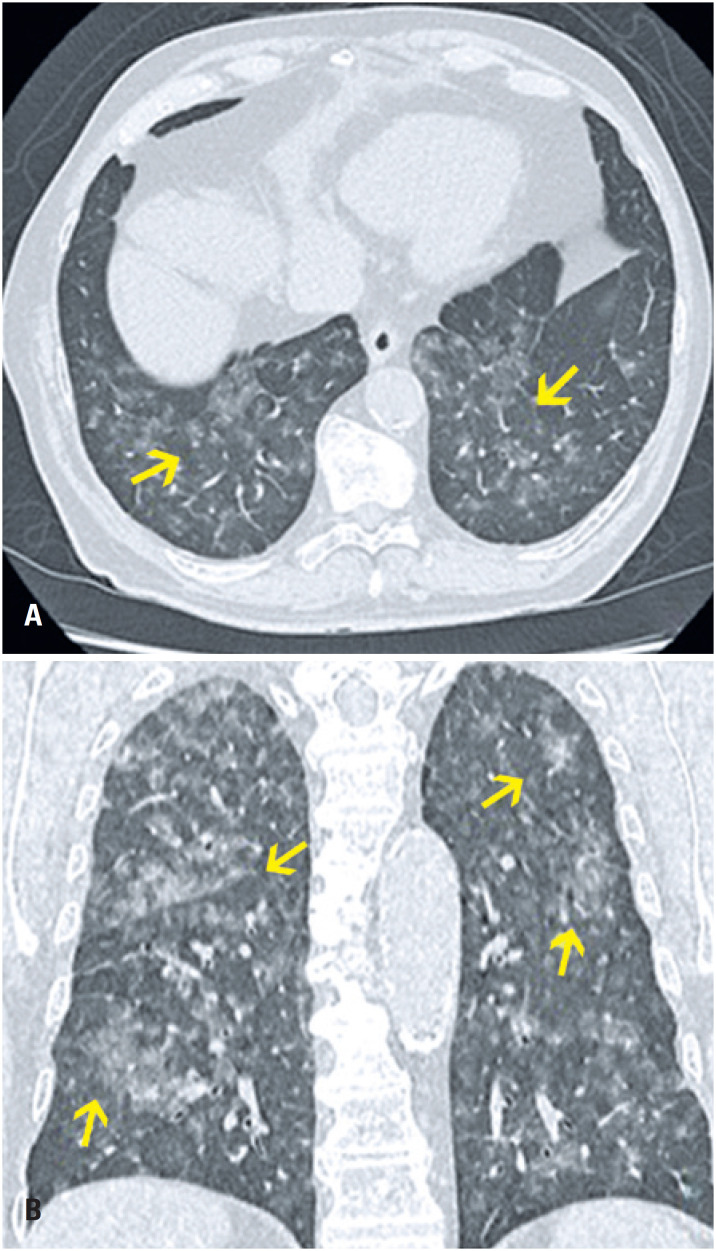
An 85-year-old male patient presented with epistaxis and hematuria for one day. He reported having had recently increased the dose of warfarin. Computed tomography in (A) Axial section and (B) Coronal reformation image: numerous centrilobular ground-glass opacities distributed in all pulmonary lobes

Alveolar hemorrhage is bleeding that originates from pulmonary microcirculation. The patient may present with hemoptysis and anemia among the clinical symptoms, and the imaging findings help narrow down the differential diagnosis. The alterations in CT include alveolar infiltrates with central distribution, sparing apices and costophrenic angles and not respecting fissures, and these infiltrates may converge in consolidations. Pulmonary involvement is usually bilateral and diffuse. In its clinical course, when the cause of the hemorrhage ceases, the pulmonary alterations tend to disappear. The definitive diagnosis is established by bronchoscopy and bronchoalveolar lavage^(^ ^11^ ^)^ ( Figure 1 ).

### Pulmonary edema

**Figure 2:**
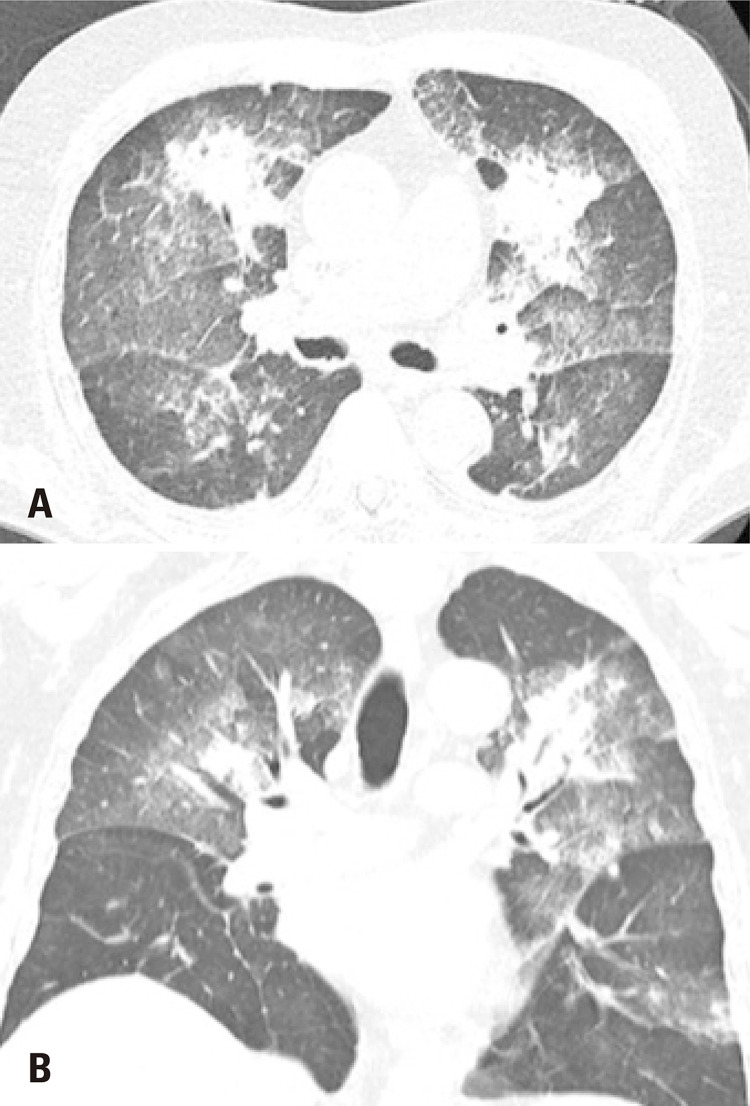
A 65-year-old male patient presented with dyspnea. Computed tomography with (A) Axial and (B) Coronal section planes: confluent ground-glass opacities and areas of alveolar consolidation with relative symmetrical distribution in the central region of both lungs, associated with diffuse smooth septal thickening and small bilateral pleural effusion, predominating on the right. Such findings are suggestive of pulmonary edema

Pulmonary edema is caused by the accumulation of fluid in the extravascular compartments, according to the capillary membrane permeability and the oncotic pressure. Radiologically, the typical aspect is the batwing sign, characterized by ground-glass opacities associated with bilateral and symmetric smooth septal thickening, sparing the periphery of the lungs.^(^ ^1^ ^,^ ^2^ ^)^ When there is an asymmetric distribution of the edema, this is probably related to mitral valve regurgitation or chronic obstructive pulmonary disease. In these cases, tomographic changes predominate, respectively, in the right upper lobe and in the regions least affected by the previous disease. When the pulmonary edema is cardiogenic, pleural effusion may be present^(^ ^7^ ^)^ ( Figure 2 ).

### Viral pneumonia

Viruses are the most common causes of respiratory infection. The imaging findings of viral pneumonias are diverse and overlap with those of non-viral infections and inflammatory conditions. Although a definitive diagnosis cannot be made with imaging findings alone, recognition of viral pneumonia patterns can help differentiate viral pathogens, and reduce the use of antibiotics.^(^ ^4^ ^,^ ^12^ ^)^

**Figure 3:**
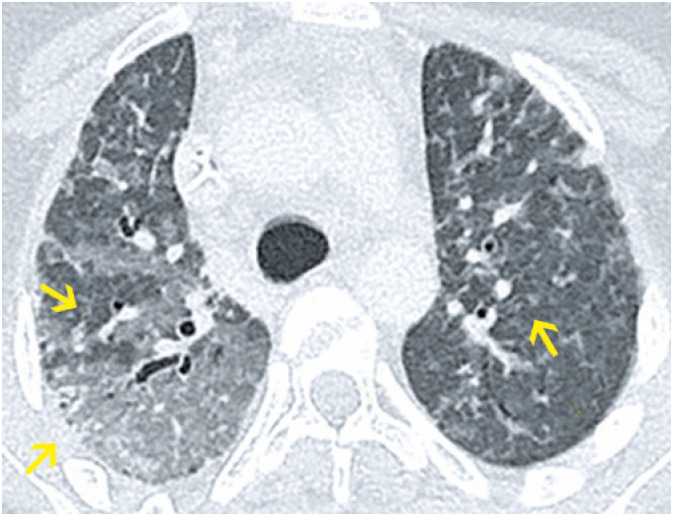
A 15-year-old female patient presented with sudden dyspnea. Personal medical history: immunosuppression. Computed tomography (axial): ground-glass opacities and bilateral peribronchovascular micronodules, accompanied by small foci of irregular consolidation. Diagnosis confirmed by bronchoalveolar lavage

Cytomegalovirus usually causes an asymptomatic infection or mild flu-like symptoms in immunocompetent patients. However, it can cause life-threatening lung infection in immunocompromised patients. The predominant radiological findings are bilateral and asymmetric ground-glass opacities, small, poorly defined centrilobular nodules, and alveolar consolidations. Thickening of interlobular septa may also be associated with this condition^(^ ^4^ ^)^ ( Figure 3 ).

**Figure 4:**
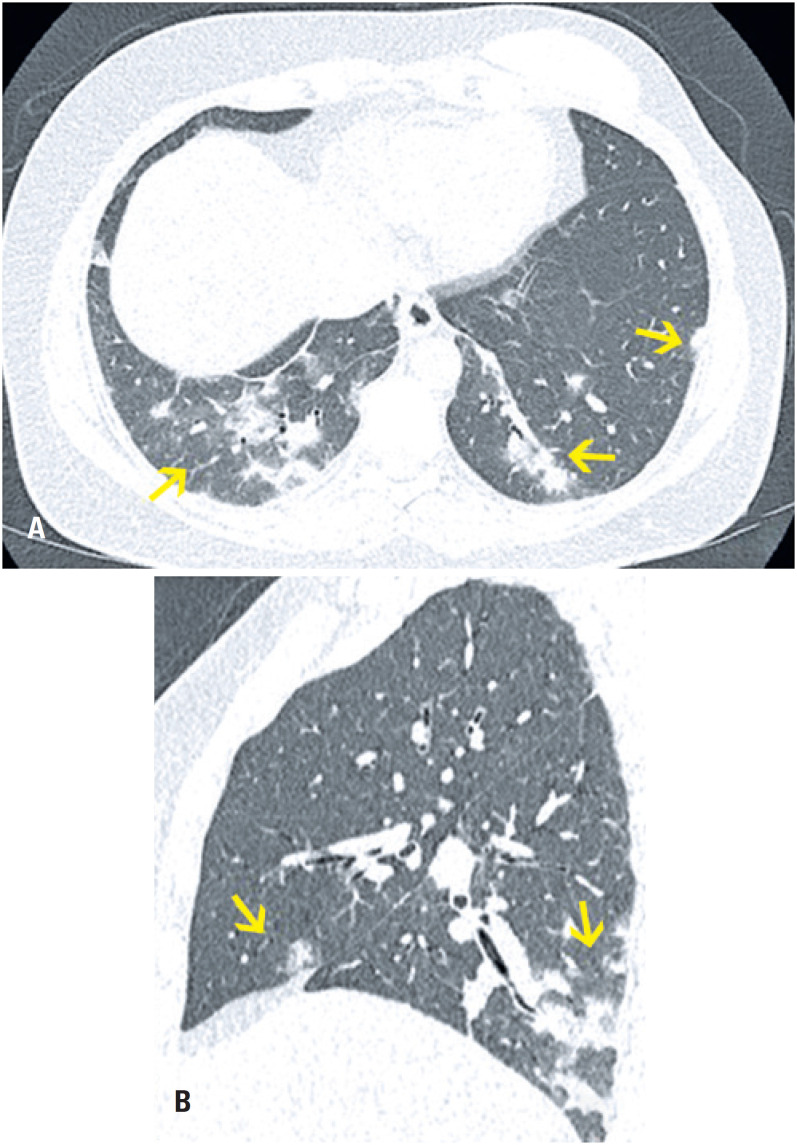
A 47-year-old female patient presented with a productive cough and myalgia. Computed tomography (A) Axial section and (B) Sagittal reformation image: micronodules and ground-glass opacities in both lungs, especially in the lower lobes, where they converge in foci of alveolar consolidation, some with a grossly nodular aspect. Respiratory C-reactive protein positive for influenza virus

The influenza virus (H1N1) causes seasonal infections of the respiratory tract, including trachea and bronchi. These infections can be periodic, endemic or pandemic. The main tomographic findings are unilateral or bilateral ground-glass opacities, which may be associated with focal or multifocal consolidation areas. These changes have a predominantly peribronchovascular and subpleural distribution, with an aspect similar to organizing pneumonia^(^ ^5^ ^)^ ( Figure 4 ).

**Figure 5:**
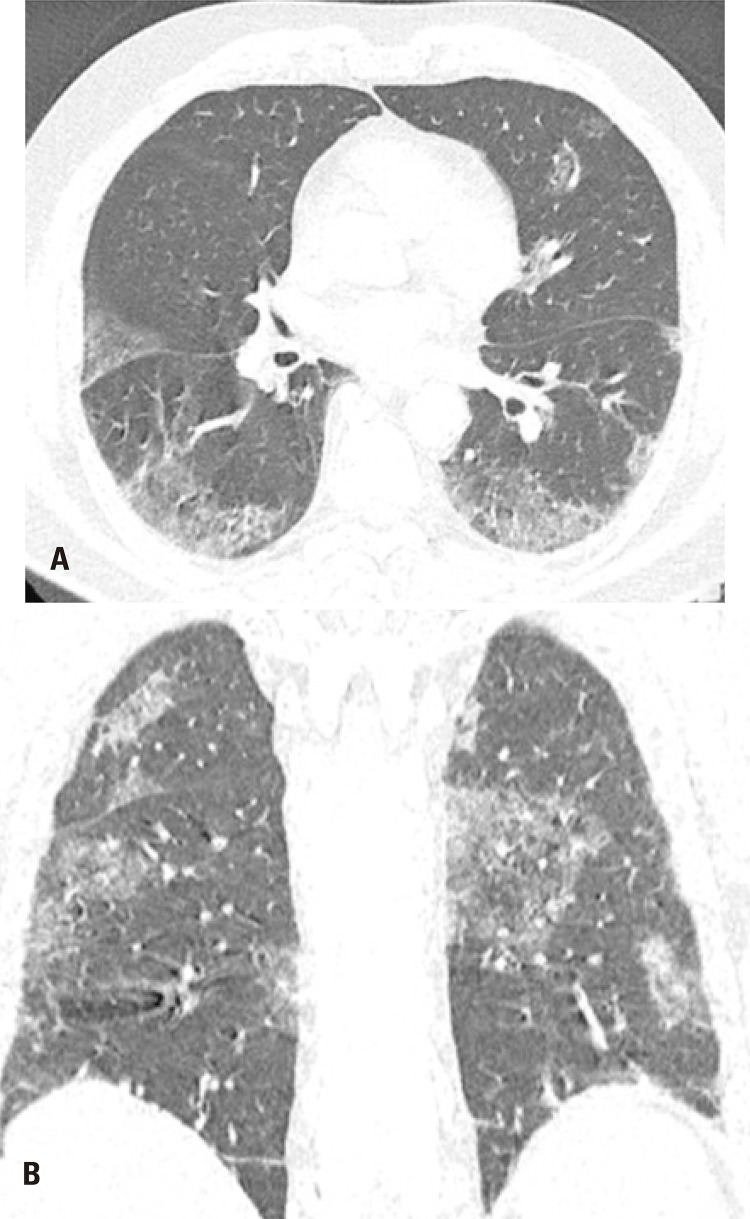
A 68-year-old male patient presented with dry cough, fever, myalgia and dyspnea. Computed tomography (A) Axial section and (B) Coronal reformation image: multiple ground-glass pulmonary opacities, sometimes associated with thickening of interlobular septa and intermingled reticulate, in multifocal, bilateral, predominantly peripheral and posterior distribution

COVID-19 has become increasingly prevalent worldwide and it reached a pandemic level in March 2020. Ground-glass opacities with bilateral distribution, with or without consolidations, preferably with peripheral distribution and in the posterior segments, are the hallmarks of COVID-19. Other CT imaging findings are crazy paving, reversed halo and consolidation foci^(^ ^10^ ^)^ ( Figure 5 ).

**Figure 6:**
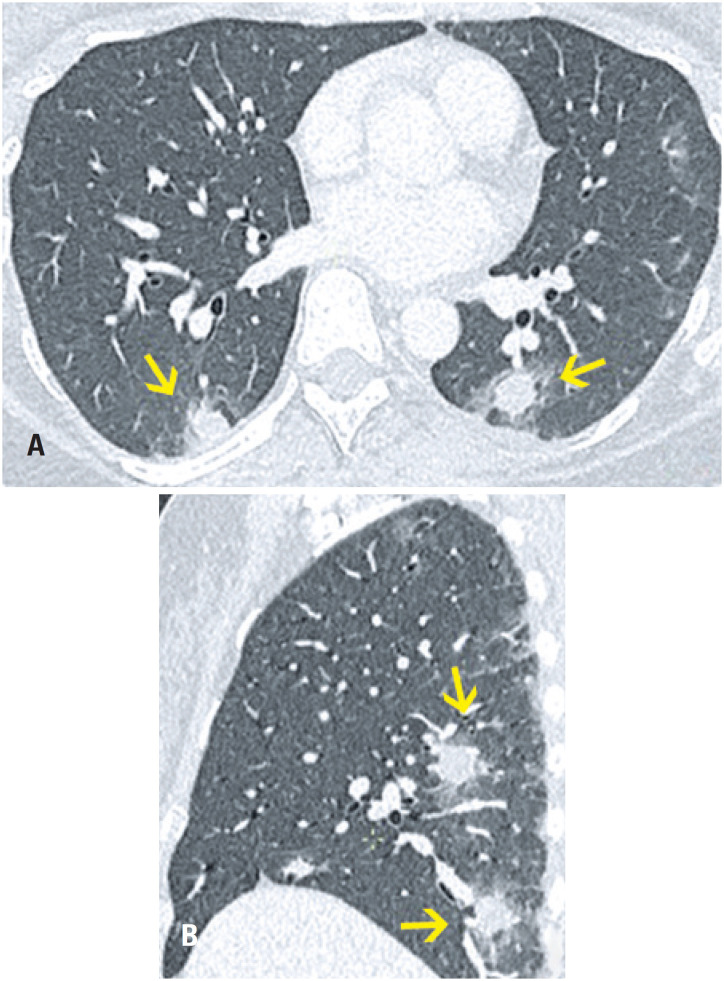
A 39-year-old female patient with cough. Computed tomography (A) Axial section (B) Sagittal reformation image: multiple pulmonary nodules with a ground-glass halo spread through the lungs, larger and more numerous in the middle and lower fields. Confirmed using measles serology

Measles is a cause of infection mainly in childhood. Even with active immunization, a significant number of older patients develop the disease, probably due to vaccine failure or non-immunization and late exposure in adulthood. Tomographic findings of measles pneumonia include ground-glass opacities, halo sign, alveolar consolidations and centrilobular micronodules. Increased hilar lymph nodes and pleural effusion are often associated with this condition ^(^ ^13^ ^,^ ^14^ ^)^ ( Figure 6 ).

### *Mycoplasma pneumoniae* pneumonia

Mycoplasma pneumonia is one of the most common cause of community-acquired pneumonia in young adults. Histologically, it is characterized by the presence of acute cellular bronchiolitis, which can progress to bronchopneumonia.

**Figure 7:**
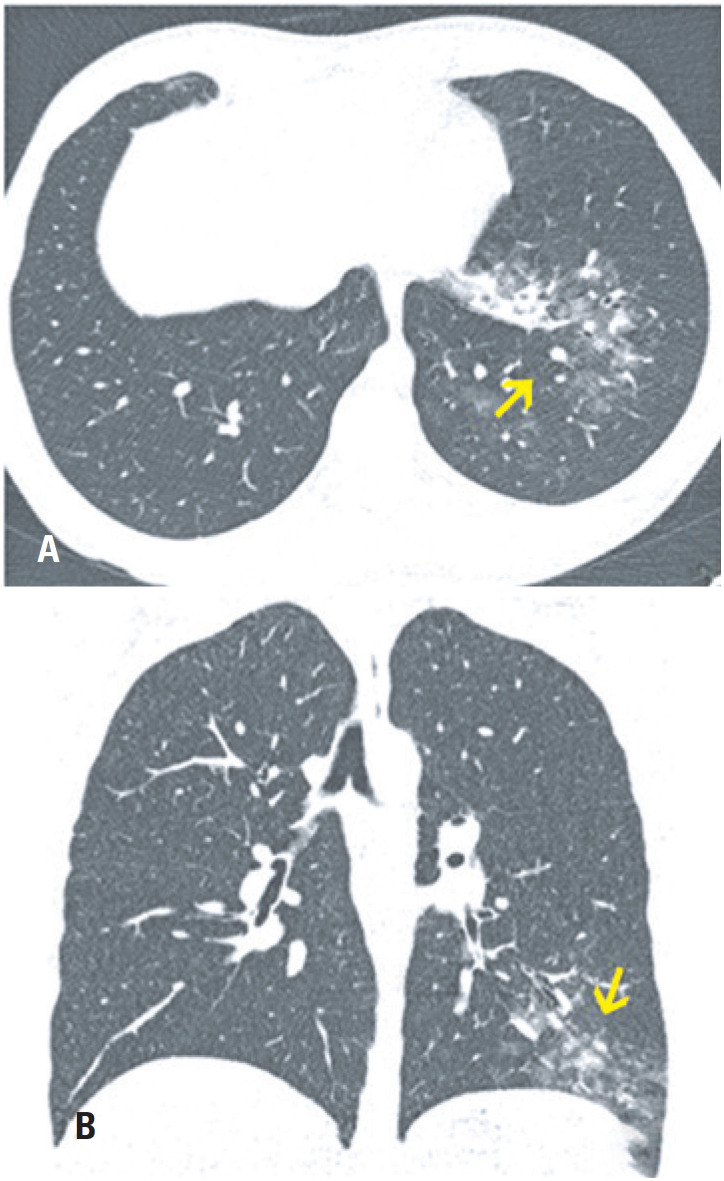
A 28-year-old male patient complaining of cough, headache and fever for 4 days. Computed tomography (A) Axial section and (B) Coronal section: thickening of bronchial walls, associated with confluent bronchocentric ground-glass opacities in the basal segments of the left lower pulmonary lobe. Such findings are compatible with a pulmonary infectious process of bronchopneumonia. Confirmed by respiratory C-reactive protein test: *Mycoplasma pneumoniae*

The most prevalent imaging findings are thickening of the peribroncovascular bundle and centrilobular nodules. Other findings are consolidations, atelectasias and ground-glass opacities^(^ ^8^ ^,^ ^15^ ^,^ ^16^ ^)^ ( Figure 7 ).

### Pulmonary infarction

**Figure 8:**
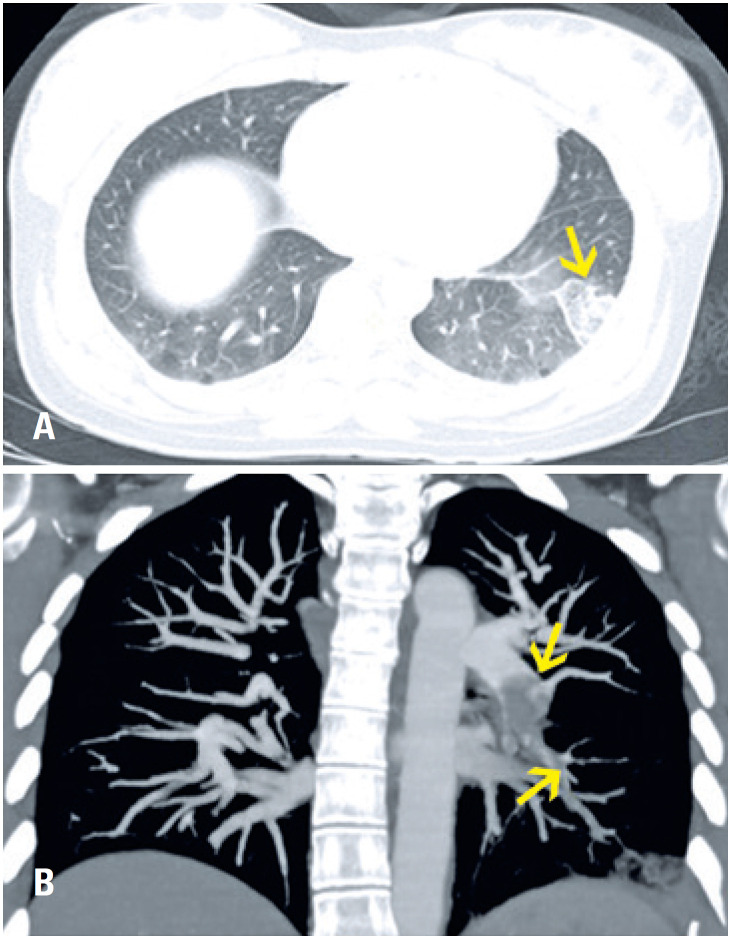
A 30-year-old woman complaining of sudden dyspnea. Computed tomography (A) Axial section and (B) Coronal reformation image with reconstruction (maximum intensity projection) of the soft tissue window: positive computed tomography angiography for pulmonary thromboembolism, with filling defects in the left interlobar artery and the segmental branches for the left lower lobe. Small reversed halo opacity. The opacity is wedge-shaped, with the apex facing the subpleural hilum in the lateral basal segment of the left lower lobe, which may correspond to the area of pulmonary infarction

Pulmonary thromboembolism is the main cause of pulmonary infarction. Its diagnosis, during the acute phase, follows a protocol with laboratory and imaging tests. A computed tomography angiography using a dedicated protocol is the gold standard imaging diagnostic method, in which a filling failure is observed in the pulmonary artery and/or its branches. The findings in a pulmonary parenchyma infarction include an reversed halo opacity (peripheral consolidation and a ground-glass opacity center), which is wedge-shaped, with the base facing the pleura and the apex facing the hilum^(^ ^17^ ^,^ ^18^ ^)^ ( Figure 8 ).

### Fat embolism

**Figure 9:**
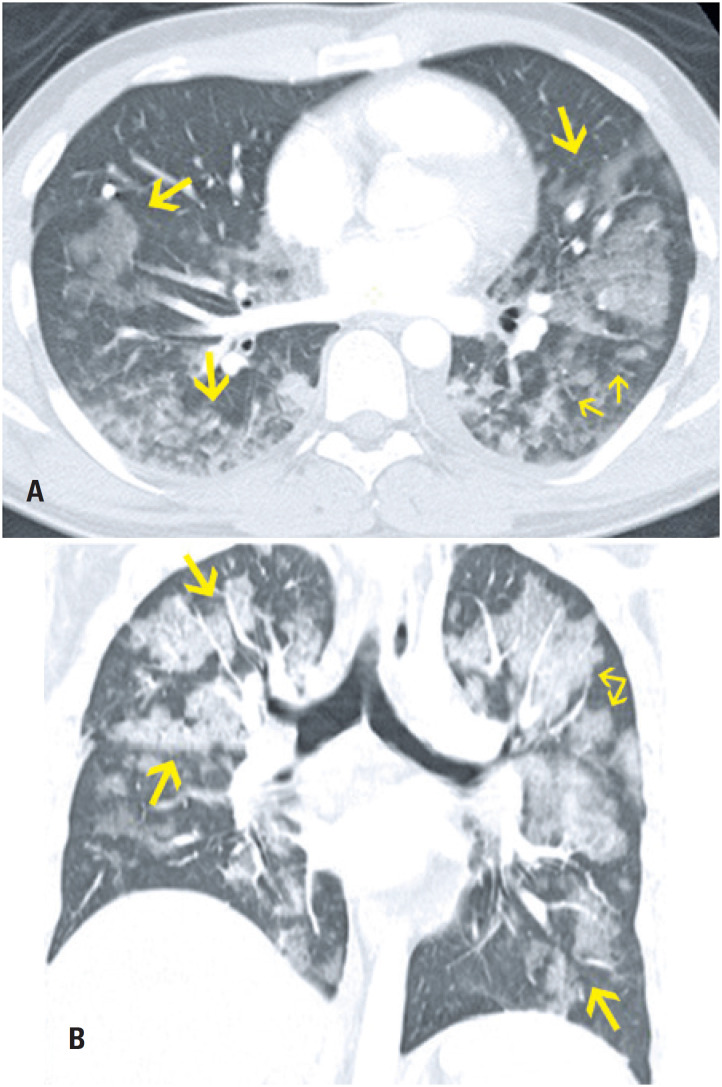
A 26-year-old male patient presented with sudden dyspnea after surgical correction of a tibial fracture using an external fixator. Computed tomography (A) Axial section and (B) Coronal reformation image: extensive ground-glass opacities and confluent alveolar consolidations predominating in the central regions of both lungs, compatible with fat embolism in the clinical context

Fat embolism results in capillaritis induced by fat globules released into the blood after long bone trauma. It manifests as well-demarcated bilateral ground-glass opacities or as poorly defined centrilobular nodules^(^ ^18^ ^)^ ( Figure 9 ).

### Pulmonary contusion

**Figure 10:**
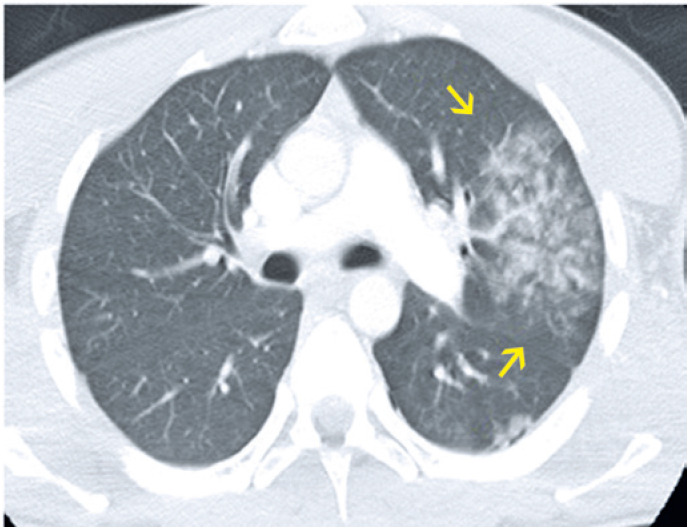
A 19-year-old man had been in a motorcycle accident. Computed tomography shows foci of consolidations located in the upper left lobe and, to a lesser extent, in the lower lobe on the same side. These findings are compatible with pulmonary contusions within the clinical context

In cases of chest trauma, chest radiography is the first exam performed to assess pneumothorax. In negative cases, further investigation is recommended using chest CT, which has greater sensitivity and specificity for all other changes related to trauma. Pulmonary contusion is the most common lung injury in the context of non-penetrating chest trauma. It is defined as a traumatic lesion in the alveoli with alveolar hemorrhage, but with no significant alveolar rupture. It appears on tomography in 6 hours after the event, and resolution begins within 24 to 48 hours. Chest CT is characterized by ground-glass opacities and/or irregular consolidations, which tend to spare the periphery of the parenchyma close to the pleura (subpleural sparing), located at the site of the trauma or in the opposite side (contrecoup injury), not respecting fissures and/or bronchial distribution^(^ ^19^ ^)^ ( Figure 10 ).

### Lipoid pneumonia

**Figure 11:**
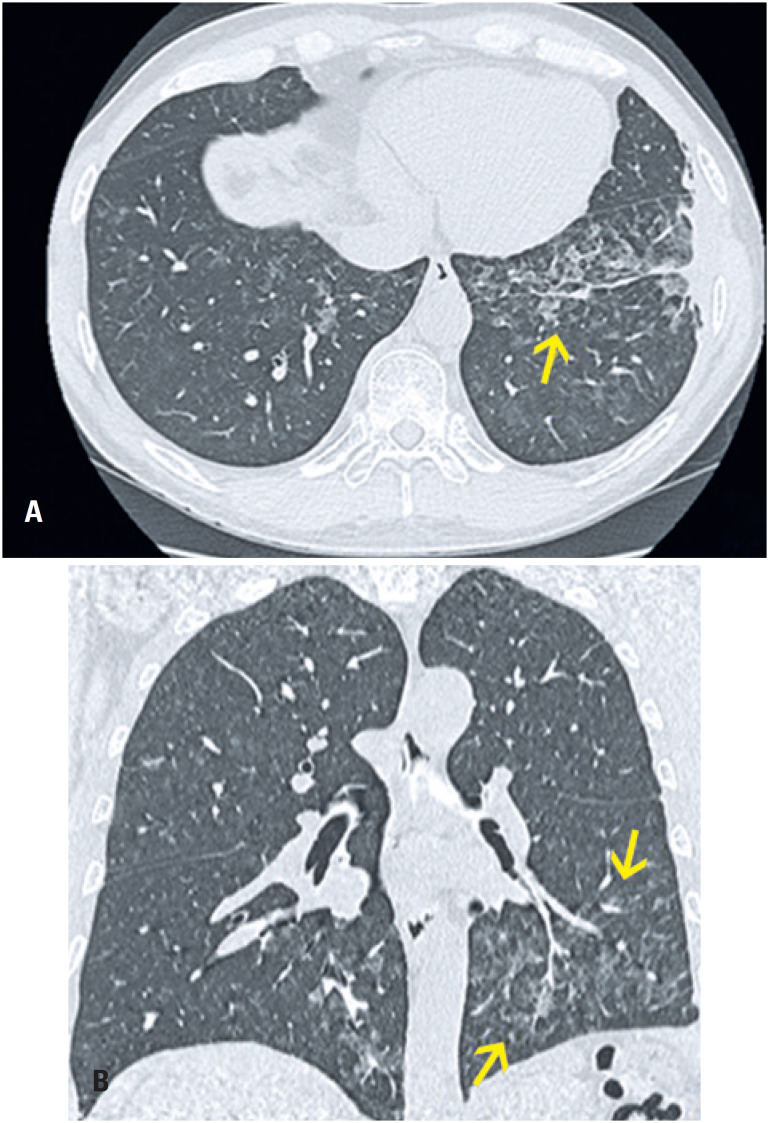
A 50-year-old asymptomatic man had the test for a medical check-up. Computed tomography (A) Axial section and (B) Coronal reformation image: multiple alveolar ground-glass opacities in both lungs, predominantly in the lower centrilobular fields. The findings were maintained in the 3-month control examination, after a biopsy was performed. The definitive diagnosis was lipoid pneumonia

Lipoid pneumonia is an uncommon condition, resulting from the suction of oils into the alveoli. In adults, the most common cause is the use of mineral oil to treat constipation, followed by the use of oily nose drops to treat chronic rhinitis. Computed tomography may show alveolar consolidations, ground-glass opacities, interlobular septum and intralobular interstitial thickening, and poorly defined centrilobular micronodules. Crazy paving pattern, which consists of thickening of interlobular septa overlaid with ground-glass opacities, is frequently observed. The most characteristic sign of lipoid pneumonia is the presence of pulmonary consolidations with fat attenuation (negative attenuation values)^(^ ^20^ ^)^ ( Figure 11 ).

### *Pneumocystis* pneumonia

**Figure 12:**
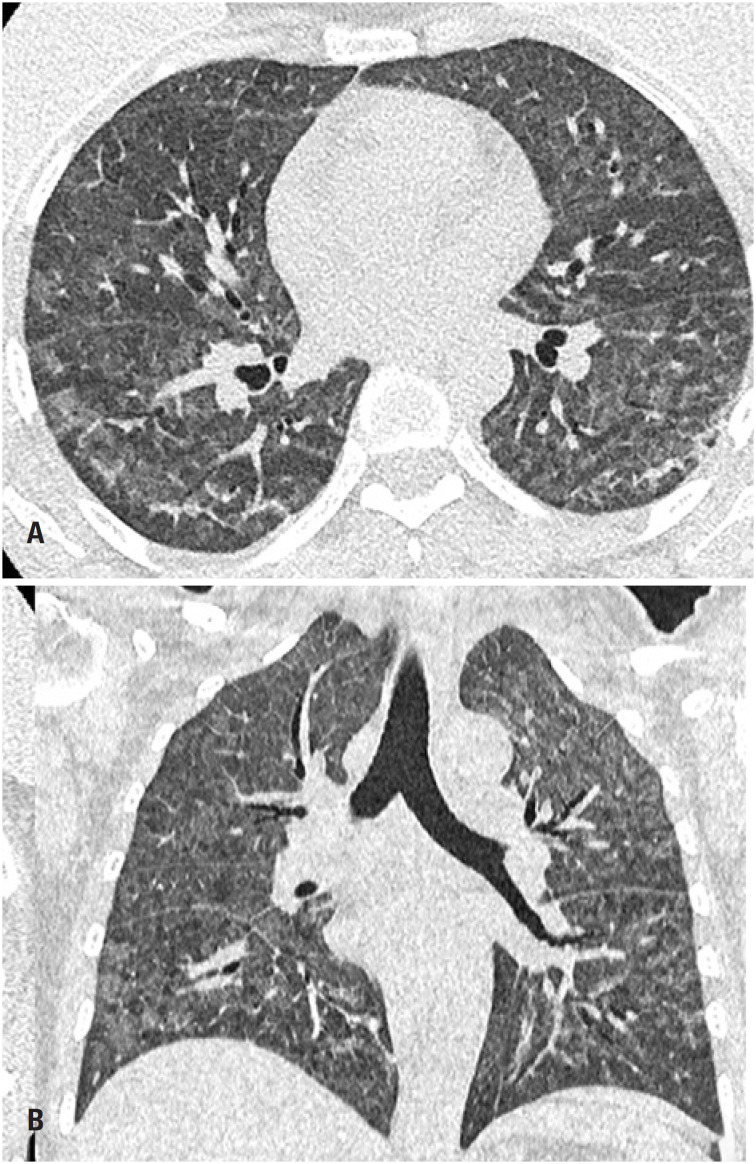
A 46-year-old woman presented with productive cough. She reported history of AIDS. Computed tomography (A) Axial section and (B) Coronal reformation image: extensive opacities affecting the pulmonary fields diffusely, with a heterogeneous ground-glass pattern associated with slight septal thickening. There was no pulmonary consolidation

It is caused by *Pneumocystis jirovecii* , an atypical fungus. It is not commonly found in the lungs of immunocompetent individuals. It is considered an opportunistic infection in immunosuppressed patients, such as patients with HIV/AIDS, cancer, undergoing chemotherapy or in chronic use of corticosteroids. On computed tomography, the main findings are extensive ground-glass opacities, usually with central distribution, relatively sparing the periphery, and there may also be a crazy paving with diffuse distribution. In more advanced disease stages, intralobular lines overlapping ground-glass opacities (mosaic pavement) and consolidation may occur. Pulmonary cysts with thick walls, of different shapes and sizes, are observed in approximately one third of patients^(^ ^8^ ^,^ ^21^ ^)^ ( Figure 12 ).

### E-cigarette, or Vaping, product use-associated lung injury

**Figure 13:**
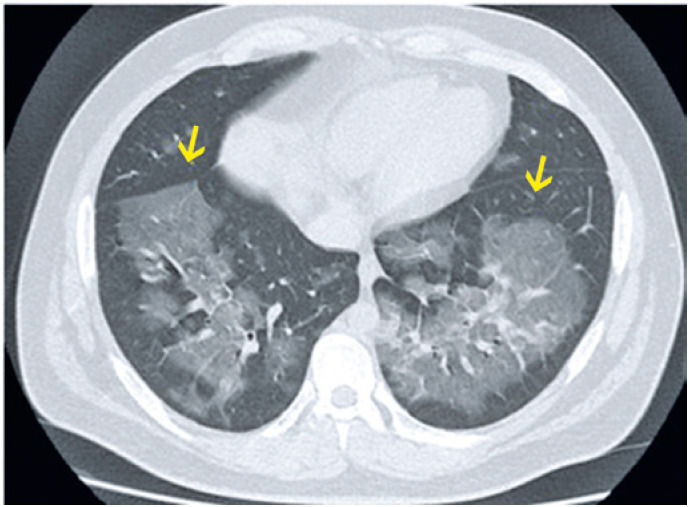
A 24-year-old male patient presented with dyspnea and cough. He reported the use of vaping. Computed tomography (axial): ground-glass opacities with diffuse peribronchovascular distribution, with predominance in the pulmonary bases, associated with thickening of the bronchial walls and sometimes thickening of the interlobular septa, in addition to small areas of consolidation in the bases

Electronic cigarettes are devices that aerosolize nicotine concentrates mixed with other solvents and are sold as an alternative to cigarettes. They have become increasingly popular, but they are not innocuous substitutes for traditional cigarettes. Several patterns of lung injury associated with vaping inhalation (Evali, standing for E-cigarette, or Vaping, product use-associated lung injury) have been described. There are several forms of radiological presentation, including hypersensitivity pneumonia, with ground-glass opacity findings, poorly defined centrilobular nodules and, occasionally, mosaic attenuation with predominance in the middle and upper lung fields. In addition, it may present as diffuse alveolar hemorrhage with centrilobular nodules, ground-glass opacities and consolidations, sparing the subpleural region; as acute lung injury, with ground-glass opacities, consolidations, and crazy paving, frequently in a gravity-dependent distribution in the acute phase; as organizing pneumonia characterized by ground-glass opacities, consolidations with peripheral or perilobular distribution, and an reversed halo sign, as well as lipoid pneumonia with ground-glass opacities, consolidations, and crazy paving, with fat attenuation in consolidations^(^ ^22^ ^)^ ( Figure 13 ).

### Organizing pneumonia

**Figure 14:**
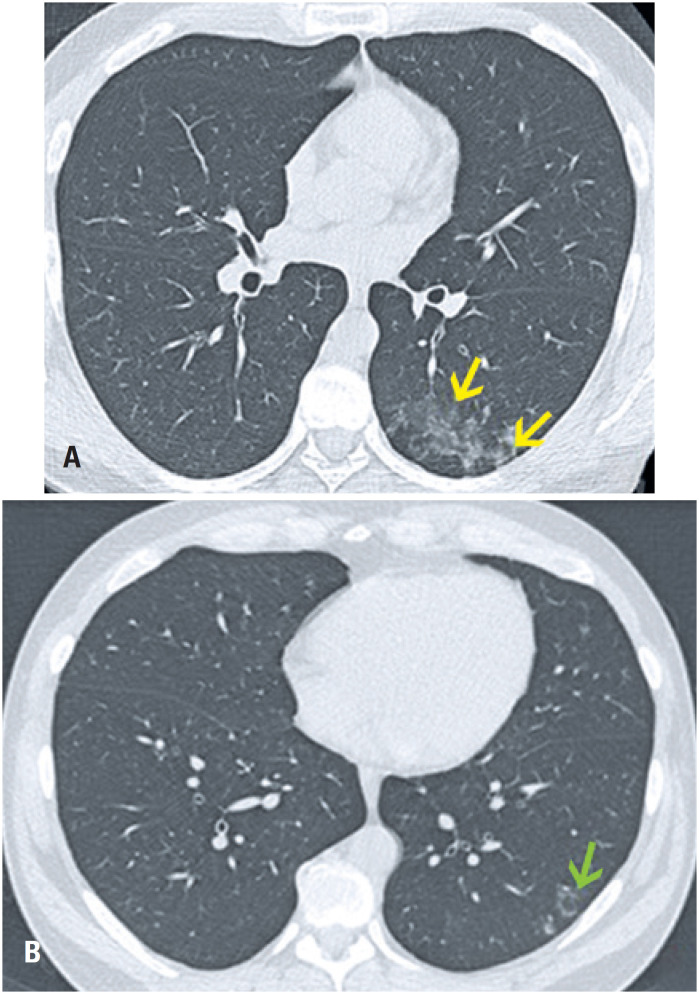
A 44-year-old male smoker had the initial exam (A) For a medical check-up. After 10 months, he had another exam (B) Due to cough and malaise. (A) Computed tomography with axial section of the pulmonary window: centrilobular ground-glass opacities, sometimes confluent, sparsely distributed in the lower left lobe; (B) Computed tomography with axial section of the pulmonary window: density reduction of centrilobular ground-glass opacities - some showing an reversed halo sign (green arrow)

The histological pattern is characterized by granulation tissue buds inside the alveoli and alveolar ducts, with chronic inflammation of the adjacent parenchyma. When it has no known cause, it is classified as primary or cryptogenic. When a causal relation can be established, it is classified as secondary. The causes are numerous and include infections, iatrogenic causes (reactions to drugs or radiation therapy), use of illicit drugs, and autoimmune diseases. Typical CT findings include ground-glass opacities, consolidations, and reversed halo images with peribronchovascular distribution^(^ ^23^ ^)^ ( Figure 14 ).

## CONCLUSION

Ground-glass opacities on chest computed tomography are very common and, for the most part, nonspecific. Therefore, for an accurate diagnosis, it is necessary to correlate the patient's clinical and laboratory data with other computed tomography imaging findings.
