# Supplementary material for: Differential diagnoses of acute ground-glass opacity in chest computed tomography: pictorial essay
Source: Einstein (Sao Paulo). 2021 Mar 5;19:eRW5772. doi: 10.31744/einstein_journal/2021RW5772 (PMC7935089; doi:10.31744/einstein_journal/2021RW5772)
Supplement: Translation

# Translation: Diagnósticos diferenciais de opacidade em vidro fosco aguda na tomografia computadorizada de tórax: ensaio pictórico

**Authors:** Marina Justi Rosa de Matos, Marcela Emer Egypto Rosa, Vanessa Mizubuti Brito, Lucas Tadashi Wada Amaral, Gabriel Laverdi Beraldo, Eduardo Kaiser Ururahy Nunes Fonseca, Rodrigo Caruso Chate, Rodrigo Bastos Duarte Passos, Murilo Marques Almeida Silva, Patrícia Yokoo, Roberto Sasdelli, Gustavo Borges da Silva Teles, Marina Carolina Bueno da Silva, Gilberto Szarf

**Affiliations:** 1 São PauloSP Brasil Hospital Israelita Albert Einstein, São Paulo, SP, Brasil.

**Keywords:** Infecções por coronavírus, COVID-19, Pandemia, SARS-CoV-2, Tórax/diagnóstico por imagem, Tomografia computadorizada por raios X, Diagnóstico diferencial

## Abstract

A opacidade em vidro fosco é uma alteração muito frequente e pouco específica na tomografia computadorizada de tórax. Ela admite grande leque de diagnósticos diferenciais no contexto agudo, desde pneumonias virais, como as causadas pelo vírus influenza, pela doença do coronavírus 2019 e pelo citomegalovírus, até mesmo lesões de origem não infecciosa, como *vaping* , infarto pulmonar, hemorragia alveolar e edema pulmonar. Para essa diferenciação diagnóstica, deve-se correlacionar o vidro fosco com os demais achados nos exames de imagem, exames laboratoriais e quadro clínico do paciente. É de suma importância, no contexto de pandemia, recordar as demais patologias com os achados semelhantes aos da doença do coronavírus 2019 nos exames de imagem.

## INTRODUÇÃO

O padrão tomográfico de vidro fosco é um achado inespecífico, podendo representar espessamento do interstício, preenchimento parcial dos alvéolos ou colapso parcial deles, aumento do aporte sanguíneo ou mesmo a associação desses achados. Radiograficamente, é definido como aumento da densidade do parênquima pulmonar, porém com preservação das marcas broncovasculares, o que difere da consolidação.^(^ ^1^ ^,^ ^2^ ^)^

As causas de opacidades em vidro fosco podem ser divididas em agudas e crônicas. Dentre as causas agudas destacam-se as infecções (virais e bacterianas atípicas), a hemorragia alveolar, o edema pulmonar, o dano alveolar difuso, a embolia pulmonar e algumas neoplasias.^(^ ^3^ ^-^ ^9^ ^)^

No contexto da pandemia causada pelo coronavírus da síndrome respiratória aguda grave 2 (SARS-CoV-2), temos a necessidade de seu diagnóstico precoce, reduzindo o risco de contágio e sua disseminação.

Apesar de ser um achado frequente na doença pelo coronavírus 2019 (COVID-19), o vidro fosco não é patognomônico da infecção pelo coronavírus. Dessa forma, não se devem negligenciar eventuais diagnósticos diferenciais. Para um diagnóstico sugestivo a partir da tomografia computadorizada (TC), deve-se correlacionar esse achado com as demais características da imagem, bem como com os dados clínicos e laboratoriais de cada paciente.^(^ ^10^ ^)^O objetivo deste trabalho foi relembrar e auxiliar na diferenciação dessas patologias, que possuem em comum a opacidade em vidro fosco no parênquima pulmonar na TC, por meio de revisão da literatura baseada em imagens tomográficas do nosso serviço.

## DIAGNÓSTICOS DIFERENCIAIS DAS OPACIDADES EM VIDRO FOSCO

### Hemorragia alveolar

Corresponde a sangramento com origem na microvasculatura pulmonar. O paciente pode apresentar hemoptise e anemia entre os sintomas clínicos, que ajudam a estreitar o diagnóstico diferencial diante dos achados de imagem. Dentre as alterações na TC, caracteriza-se o infiltrado alveolar com distribuição central, ápices e seios costofrênicos são poupados e não se respeitam fissuras, podendo confluir em consolidações. O acometimento pulmonar geralmente é bilateral e difuso. Em sua evolução, desde que cessada a causa da hemorragia, as alterações pulmonares tendem a desaparecer. O diagnóstico definitivo é realizado por broncoscopia e lavado broncoalveolar^(^ ^11^ ^)^( Figura 1 ).

**Figura 1:** Homem, 85 anos, apresentando epistaxe e hematúria há 1 dia. Refere ter aumentado a dose do medicamento varfarina recentemente. Tomografia computadorizada (A) Axial (B) Reformatação coronal: numerosas opacidades em vidro fosco centrolobulares distribuídas por todos os lobos pulmonares

### Edema pulmonar

O edema pulmonar é causado pelo acúmulo de líquido nos compartimentos extravasculares, conforme a permeabilidade da membrana do capilar e da pressão oncótica. Radiologicamente, o aspecto típico é o de “asa de borboleta”, caracterizado por opacidades em vidro fosco associadas a espessamento liso dos septos interlobulares, de forma bilateral e simétrica, poupando a periferia dos pulmões.^(^ ^1^ ^,^ ^2^ ^)^Quando há distribuição assimétrica do edema, este provavelmente está relacionado à regurgitação da valva mitral ou à doença pulmonar obstrutiva crônica. Nesses casos, as alterações tomográficas predominam, respectivamente, no lobo superior direito e nas regiões menos afetadas pela doença prévia. Quando a causa do edema pulmonar for de origem cardíaca, derrame pleural pode estar presente^(^ ^7^ ^)^( Figura 2 ).

**Figura 2:** Homem, 65 anos, com quadro de dispneia. Tomografia computadorizada com plano de corte (A) Axial (B) Coronal: opacidades em vidro fosco confluentes e áreas de consolidação alveolar distribuídas de forma relativamente simétrica na região central de ambos os pulmões, associadas a espessamento septal liso difuso e pequeno derrame pleural bilateral, predominando à direita. Tais achados são sugestivos de edema pulmonar

### Pneumonias virais

Os vírus são as causas mais comum de infecção respiratória. Os achados de imagem das pneumonias virais são diversos e se sobrepõem aos de infecções não virais e condições inflamatórias. Embora um diagnóstico definitivo não possa ser dado apenas com os achados de imagem, o reconhecimento dos padrões de pneumonia viral podem ajudar a diferenciar os patógenos virais e reduzir o uso de antibióticos.^(^ ^4^ ^,^ ^12^ ^)^

O citomegalovírus geralmente causa uma infecção assintomática ou com sintomas gripais leves em pacientes imunocompetentes. Entretanto pode causar infecção pulmonar ameaçadora à vida em pacientes imunodeprimidos. Os achados radiológicos predominantes são opacidades em vidro fosco bilaterais e assimétricas, pequenos nódulos centrolobulares mal definidos e consolidações alveolares. Espessamento dos septos interlobulares também pode estar associado^(^ ^4^ ^)^( Figura 3 ).

**Figura 3:** Paciente do sexo feminino, 15 anos, com quadro de dispneia súbita. Antecedente pessoal: imunossuprimida. Tomografia computadorizada (axial): opacidades em vidro fosco e de micronódulos peribroncovasculares bilaterais, acompanhadas de pequenos focos de consolidação irregular. Diagnóstico confirmado por lavado broncoalveolar

O vírus influenza (H1N1) causa infecções sazonais do trato respiratório, incluindo traqueia e brônquios fontes. Essas infecções podem ser periódicas, endêmicas ou pandêmicas. Os principais achados tomográficos são opacidades em vidro fosco unilaterais ou bilaterais, podendo se associar a áreas focais ou multifocais de consolidação. Essas alterações têm distribuição predominantemente peribroncovascular e subpleural, com aspecto semelhante à pneumonia em organização^(^ ^5^ ^)^( Figura 4 ).

**Figura 4:** Paciente do sexo feminino, 47 anos, com queixa de tosse produtiva e mialgia. Tomografia computadorizada (A) Axial (B) Reformatação sagital: micronódulos e opacidades em vidro fosco em ambos os pulmões, principalmente nos lobos inferiores, onde confluem em focos de consolidação alveolar, algumas com aspecto grosseiramente nodular. A proteína C-reativa respiratório positivo para vírus da influenza

A COVID-19 tem se tornado cada vez mais prevalente mundialmente, tendo alcançado estágio de pandemia em março de 2020. Opacidades em vidro fosco com distribuição bilateral, com ou sem consolidações, apresentando distribuição preferencialmente periférica e nos segmentos posteriores, são a marca da COVID-19. Outros achados de imagem na TC são pavimentação em mosaico, halo invertido e focos de consolidação^(^ ^10^ ^)^( Figura 5 ).

**Figura 5:** Paciente do sexo masculino, 68 anos, com queixa de tosse seca, febre, mialgia e dispneia. Tomografia computadorizada (A) Axial (B) Reformatação coronal: múltiplas opacidades pulmonares em vidro fosco, por vezes associadas a espessamento de septos interlobulares e reticulado de permeio, em distribuição multifocal, bilateral, predominantemente periférica e posterior

O sarampo é uma causa de infecção principalmente na infância. Mesmo com imunização ativa, um número significativo de pacientes mais velhos desenvolve a doença, provavelmente por falha da vacina ou não imunização e exposição tardia na idade adulta. Os achados tomográficos da pneumonia por sarampo incluem opacidades em vidro fosco, sinal do halo, consolidações alveolares e micronódulos centrolobulares. Aumento de linfonodos hilares e derrame pleural estão frequentemente associados^(^ ^13^ ^,^ ^14^ ^)^( Figura 6 ).

**Figura 6:** Paciente do sexo feminino, 39 anos, com queixa de tosse. Tomografia computadorizada (A) Axial (B) Reformatação sagital: múltiplos nódulos pulmonares com halo em vidro fosco disseminados pelos pulmões, maiores e mais numerosos nos campos médios e inferiores. Confirmado com sorologia para sarampo

### Pneumonia por *Mycoplasma pneumoniae*

Pneumonia por micoplasma é uma das causa mais comum de pneumonia adquirida na comunidade em adultos jovens. Histologicamente, é caracterizada pela presença de bronquiolite celular aguda, que pode progredir para broncopneumonia.

Os achados de imagem com maior prevalência são espessamento do feixe peribroncovascular e nódulos centrolobulares. Outros achados são consolidações, atelectasias e opacidades em vidro fosco^(^ ^8^ ^,^ ^15^ ^,^ ^16^ ^)^( Figura 7 ).

**Figura 7:** Homem, 28 anos, com queixa de tosse, cefaleia e febre há 4 dias. Tomografia computadorizada (A) Com corte axial (B) Coronal: espessamento de paredes brônquicas, associado a opacidades em vidro fosco broncocêntricas confluentes nos segmentos basais do lobo pulmonar inferior esquerdo. Tais achados são compatíveis com processo infeccioso pulmonar broncopneumônico. Confirmado por exame de proteína C-reativa respiratório: *Mycoplasma pneumoniae*

### Infarto pulmonar

O tromboembolismo pulmonar é a principal causa de infarto pulmonar. Seu diagnóstico, durante a fase aguda, segue um protocolo com exames laboratoriais e de imagem. A angiotomografia com protocolo dedicado é o método de imagem padrão-ouro, no qual se observa falha de enchimento na artéria pulmonar e/ou em seus ramos. Os achados de infarto do parênquima pulmonar propriamente dito incluem opacidade com halo invertido (consolidação periférica e centro em vidro fosco), de morfologia em cunha, com a base voltada para a pleura e o ápice voltado para o hilo^(^ ^17^ ^,^ ^18^ ^)^( Figura 8 ).

**Figura 8:** Mulher, 30 anos, com queixa de dispneia súbita. Tomografia computadorizada (A) Com corte axial (B) Reformatação coronal com reconstrução (projeção de intensidade máxima) da janela de partes moles: angiotomografia positiva para tromboembolismo pulmonar, notando-se falhas de enchimento na artéria interlobar esquerda e ramos segmentares para o lobo inferior esquerdo. Pequena opacidade em halo invertido. Em cunha com o ápice voltado para o hilo subpleural no segmento basal lateral do lobo inferior esquerdo, que pode corresponder à área de infarto pulmonar

### Embolia gordurosa

A embolia gordurosa se traduz patologicamente por uma capilarite induzida por glóbulos de gordura liberados no sangue após trauma de ossos longos. Manifesta-se como opacidades em vidro fosco bem demarcado bilaterais ou como nódulos centrolobulares mal definidos^(^ ^18^ ^)^( Figura 9 ).

**Figura 9:** Homem, 26 anos, com queixa de dispneia súbita após correção cirúrgica de fratura da tíbia com uso de fixador externo. Tomografia computadorizada (A) Com corte axial (B) Reformatação coronal: extensas opacidades em vidro fosco e consolidações alveolares confluentes predominando na região central de ambos os pulmões, compatíveis com embolia gordurosa no contexto clínico

### Contusão pulmonar

Em casos de trauma torácico, primeiro realiza-se radiografia de tórax, para o diagnóstico de pneumotórax. Nos casos negativos, recomenda-se prosseguir a investigação com TC de tórax, a qual apresenta maior sensibilidade e especificidade para todas as demais alterações relacionadas ao trauma. A contusão pulmonar é a lesão pulmonar mais comum no contexto de trauma torácico não penetrante. Representa lesão traumática nos alvéolos com hemorragia alveolar, mas sem ruptura alveolar significativa. Já possui representatividade na tomografia após 6 horas do ocorrido e início da absorção entre 24 e 48 horas. A TC de tórax é caracterizada por opacidades em vidro fosco e/ou consolidações irregulares, que tendem a poupar a periferia do parênquima próximo à pleura ( *subpleural sparing* ), localizadas no local do trauma ou na região contrária a ele (contragolpe), sem respeitar fissuras e/ou distribuição brônquica^(^ ^19^ ^)^( Figura 10 ).

**Figura 10:** Homem, 19 anos, sofreu queda de moto. Tomografia computadorizada mostra focos de consolidações localizados no lobo superior esquerdo e, em menor grau, no lobo inferior desse mesmo lado. Achados compatíveis com contusões pulmonares dentro do contexto clínico

### Pneumonia lipoídica

A pneumonia lipoídica é uma condição incomum, resultante da aspiração de óleos para os alvéolos. Em adultos, a causa mais comum é o uso de óleo mineral para tratamento de constipação, seguido do uso de gotas nasais oleosas para tratamento de rinite crônica. A TC pode evidenciar consolidações alveolares, opacidades em vidro fosco, espessamento de septos interlobulares e do interstício intralobular e micronódulos centrolobulares pouco definidos. Padrão de pavimentação em mosaico, que consiste em espessamento de septos interlobulares sobrepostos a opacidades em vidro fosco, é frequentemente observado. O sinal mais característico da pneumonia lipoídica é a presença de consolidações pulmonares com atenuação de gordura (valores de atenuação negativos)^(^ ^20^ ^)^( Figura 11 ).

**Figura 11:** Homem, 50 anos, assintomático, realiza o exame para *check-up* . Tomografia computadorizada (A) Em plano de corte axial (B) Reformatação coronal: múltiplas opacidades alveolares em vidro fosco em ambos os pulmões, predominantemente centrolobulares nos campos inferiores. Os achados se mantiveram no exame de controle em 3 meses, tendo sido realizada a biópsia, e o diagnóstico definitivo foi de pneumonia lipoídica

### Pneumonia por *Pneumocystis*

É causada por *Pneumocystis jirovecii* , um fungo atípico. Não é comumente encontrado nos pulmões de pessoas imunocompetentes. É considerada uma infecção oportunista em pacientes imunossuprimidos, como os portadores de HIV/AIDS, câncer, em quimioterapia ou em uso crônico de corticosteroides. Na TC, os principais achados são extensas opacidades em vidro fosco, geralmente com distribuição central, poupando relativamente a periferia, também podendo haver padrão em mosaico e com distribuição difusa. Na doença mais avançada, podem se desenvolver linhas intralobulares sobrepostas ao vidro fosco (pavimentação em mosaico) e consolidação. Cistos pulmonares com paredes espessadas, de diferentes formatos e tamanhos, ocorrem em cerca de um terço dos pacientes^(^ ^8^ ^,^ ^21^ ^)^( Figura 12 ).

**Figura 12:** Mulher, 46 anos, com queixa de tosse produtiva. Refere antecedente de AIDS. Tomografia computadorizada (A) Com corte axial (B) Reformatação coronal: extensas opacidades acometendo difusamente os campos pulmonares, com padrão heterogêneo em vidro fosco associado a discreto espessamento septal. Não há consolidação pulmonar

### E-cigarette, or Vaping, product use-associated lung injury

Cigarros eletrônicos são dispositivos que aerossolizam concentrados de nicotina misturados com outros solventes, sendo vendidos como alternativa aos cigarros. Eles têm se tornado cada vez mais populares, porém não se trata de um substituto inócuo ao cigarro tradicional. Diversos padrões de injúria pulmonar associada à inalação do *vaping* ((Evali - *E-cigarette, or Vaping, product use-associated lung injury* ) têm sido descritos. Há várias formas de apresentação radiológica, dentre elas a pneumonia de hipersensibilidade, com os achados de opacidades em vidro fosco, nódulos centrolobulares mal definidos e, ocasionalmente, atenuação em mosaico com predomínio nos campos pulmonares médios e superiores. Ainda, pode se apresentar como hemorragia alveolar difusa com nódulos centrolobulares, opacidades em vidro fosco e consolidações, poupando a região subpleural; como lesão pulmonar aguda, por meio de opacidades em vidro fosco, consolidações e pavimentação em mosaico, com frequente distribuição gravitacional dependente na fase aguda; como pneumonia em organização caracterizada por opacidades em vidro fosco, com consolidações com distribuição periférica ou perilobular, podendo também apresentar sinal do halo invertido e, ainda, como pneumonia lipoídica com opacidades em vidro fosco, consolidações e pavimentação em mosaico, com atenuação de gordura nas consolidações^(^ ^22^ ^)^( Figura 13 ).

**Figura 13:** Homem, 24 anos, com queixa de dispneia e tosse. Refere uso de *vaping* . Tomografia computadorizada (axial): opacidades em vidro foco de distribuição peribroncovascular difusa, com predomínio nas bases pulmonares, associadas a espessamento de paredes brônquicas e, por vezes, espessamento dos septos interlobulares, além de pequenas áreas de consolidação de permeio nas bases

### Pneumonia em organização

Corresponde a um padrão histológico caracterizado por pólipos de tecido de granulação no interior dos alvéolos e ductos alveolares, com inflamação crônica do parênquima adjacente. Quando não tem causa conhecida, é classificada como primária ou criptogênica. Quando uma relação causal pode ser estabelecida, é classificada como secundária. As causas são inúmeras e incluem infecções, causas iatrogênicas (reações a drogas ou terapia com radiação), uso de drogas ilícitas e doenças autoimunes. Os achados típico na TC incluem opacidades em vidro fosco, consolidações e imagens de halo invertido com distribuição peribroncovascular^(^ ^23^ ^)^( Figura 14 ).

**Figura 14:** Homem, 44 anos, tabagista, realizou o primeiro exame (A) Para *check-up* . Após 10 meses, realiza o segundo exame (B) Devido à tosse e a mal-estar. (A) Tomografia computadorizada com plano de corte axial da janela pulmonar: opacidades centrolobulares em vidro fosco por vezes confluentes, esparsas pelo lobo inferior esquerdo; (B) Tomografia computadorizada com plano de corte axial da janela pulmonar: redução da densidade das opacidades centrolobulares em vidro fosco – algumas exibindo sinal do halo invertido (seta verde)

## CONCLUSÃO

Opacidades em vidro fosco na tomografia computadorizada de tórax são muito comuns e, em sua maioria, inespecíficas. Portanto, para um diagnóstico preciso, é necessário correlacionar os dados clínicos e laboratoriais do paciente com os demais achados da tomografia computadorizada.
